# Ozone enhances the efficacy of radiation therapy in esophageal cancer

**DOI:** 10.1093/jrr/rrae041

**Published:** 2024-06-06

**Authors:** Jiayou Guo, Jiayi Guo, Beibei Cheng, Mengxiao Gong, Xingbang Sun, Hongwei Zhang, Jianxin Ma

**Affiliations:** Department of Oncology, Lianyungang Oriental Hospital affiliated to Xuzhou Medical University, Lianyungang 222042, China; Department of Oncology, Lianyungang Oriental Hospital affiliated to Xuzhou Medical University, Lianyungang 222042, China; Department of Oncology, Lianyungang Oriental Hospital affiliated to Xuzhou Medical University, Lianyungang 222042, China; Department of Oncology, Lianyungang Oriental Hospital affiliated to Xuzhou Medical University, Lianyungang 222042, China; Department of Oncology, Lianyungang Oriental Hospital affiliated to Xuzhou Medical University, Lianyungang 222042, China; Department of Oncology, Lianyungang Oriental Hospital affiliated to Xuzhou Medical University, Lianyungang 222042, China; Department of Oncology, Lianyungang Oriental Hospital affiliated to Xuzhou Medical University, Lianyungang 222042, China

**Keywords:** ozone, neutrophil extracellular traps, esophageal cancer, radio resistance, AMPK, scavenger receptor-A

## Abstract

Radioresistance is increasingly developed in esophageal cancer. Increasing radiation sensitivity can reduce the mortality of esophageal cancer. To investigate the effect and mechanism of ozone on the radiotherapy sensitization of esophageal carcinoma. KYSE150 cells were xenografted subcutaneously into nude mice and irradiated with 8 Gy radiation according to different subgroups (sham, radiation, ozone and radiation+ozone group (*n* = 10 per group)). Half of the mice were used to determine the body weight, tumor size and tumor weight. Half of the mice were used to collect peripheral blood. The serum was centrifuged to detect circulating cell-free DNA (cf-DNA), interleukin-6 (IL-6), interferon-γ (IFN-γ), myeloperoxidase (MPO)-DNA complexes, tumor necrosis factor-α (TNF-α), matrix metalloproteinase-9 (MMP-9) and hypoxia-inducible factor-1α (HIF-1α) using commercial kits. The levels of phosphorylation AMP-activated protein kinase (p-AMPK) and scavenger receptor-A (SR-A) were measured by immunocytochemistry and Western blotting in the tumor tissues of mice. Ozone alone or combined with radiation therapy significantly reduced the body weight, tumor volume and tumor weight of esophageal cancer compared to the sham group. The ELISA results showed that the levels of cf-DNA, IFN-γ, MPO-DNA complexes, TNF-α, IL-6, HIF-1α and MMP-9 in the peripheral blood of mice treated with ozone combined with radiation were significantly lower compared with the radiation group. Ozone, synergistically with radiation, significantly increased the protein expression of p-AMPK and SR-A. Ozone may increase the radiosensitivity of esophageal cancer by inhibiting neutrophil extracellular traps.

## INTRODUCTION

In recent years, a gradual increase in the incidence rate has occurred in esophageal cancer [1]. It is estimated that there will be 957 000 new cases and 880 000 deaths by 2040 [2]. China accounts for ~54% of global diagnosed cases of esophageal cancer [1]. Surgery remains the preferred therapy for early esophageal squamous cell carcinoma patients. However, this cancer is usually prone to missed diagnosis early due to the unspecific symptoms, resulting in a 5-year survival rate of <20% [3]. For patients with advanced esophageal cancer, radiotherapy is the current standard treatment method, especially for esophageal squamous cell carcinoma [4]. However, the most important factor currently limiting the effectiveness of radiotherapy for advanced esophageal cancer is the formation of radiation-resistant cancer cells after repeated irradiation [5]. This makes radiotherapy unable to substantially improve the prognosis and long-term survival of esophageal cancer patients. Therefore, urgent needs are emerging to find new adjuvant treatments and mechanisms to prevent the development of radiation resistance.

Hypoxia and chronic inflammation are the core mechanisms leading to tumor progression and radiation resistance [6]. In advanced cancer, apoptosis, necrosis, and the release of numerous damage-associated molecular patterns (DAMPs) can induce activation of Toll-like receptor 4 (TLR4) in neutrophils and lead to the release of neutrophil nuclear contents, forming neutrophil extracellular trapping networks (NETs) [7]. NETs are a novel form of death that differs from apoptosis or even necrosis [8]. Instead, NETs play an important role in causing hypoxia and chronic inflammation in the tumor microenvironment [9]. NETs promote platelet adhesion, aggregation, activation and fibrin deposition, and initiate thrombus formation, leading to cell hypoxia [10]. Hypoxia can activate hypoxia-inducible factor 1α (HIF-1α), which is related to cellular activities, such as glucose metabolism balance, antioxidant factor release, vascular protection and regeneration [11]. Hypoxia enables cancer cells to acquire the ability to resist radiation [12]. Moreover, NETs can activate TLR4/9-COX2 signaling and then trigger tumor-related inflammation, cell death resistance or enhanced invasiveness or metastasis [13]. The pretreatment of inhibiting the adaptation of cancer cells to hypoxia is a promising method for resisting radiation resistance.

Medical ozone has the function of killing bacteria, promoting oxygen metabolism and activating the immune system [14]. It has been used to regulate inflammation and applied to treating wound healing, ischemic diseases, infections and chronic inflammatory diseases. Also, ozone has been found to be effective in the treatment of lumbar disc herniation, arthralgia, diabetes ulcers and chronic ulcerative colitis [15]. Previous data has indicated that ozone could protect animals from radiation-induced organ toxicity by increasing the endogenous antioxidant defense mechanism [16]. Ozonized oil nanoemulsions have been identified as radiosensitizers in B-16 melanoma and OV-90 ovarian cells *in vitro* [17]. The high-mobility group box 1 (HMGB1) protein is one of the core components of NETs [18]. Mechanically, ozone can upregulate the phagocytic effect of scavenger receptor A (SR-A) on HMGB1 by activating adenosine monophosphate-activated protein kinase (AMPK) and can also stimulate the inflammatory response by being recognized by inflammatory cells as DAMPs [19]. Therefore, ozone can reduce the activation of NETs at the source by reducing the already-formed NETs and by phagocytosing DAMPs, thus potentially becoming an effective sensitizer for tumor radiotherapy.

The purpose of this study was to investigate the potential sensitization effect of ozone in tumor radiotherapy. Here, we established a mouse xenograft tumor model of esophageal cancer to explore the potential mechanism of ozone-enhancing radio sensitization *in vivo*.

## MATERIALS AND METHODS

### Reagents and materials

The esophageal cancer KYSE150 (#TCHu236) cell line was provided by the cell bank of the Chinese Academy of Sciences (Shanghai, China); PicoGreen® DsDNA Reagent (#P7589) is provided by Invitrogen (Life Technologies, Carlsbad, CA, USA). Ozone is generated by medical ozone treatment equipment (Ozomed Smart, Kastner-Praxisbedarf GmbH, Rastatt, Germany). Interleukin-6 (IL-6, #PD6050), interferon-γ (INF-γ, #QK285), tumor necrosis factor-α (TNF-α, #STA00D), matrix metalloproteinase-9 (MMP-9, #SMP900) and HIF-1α (#DYC1935-5) enzyme-linked immunosorbent assay kits were produced by R&D Systems (Minneapolis, USA). HIF-1α antibody (#3716) was purchased from Cell Signaling Technology (Danvers, USA). Roswell Park Memorial Institute (RPMI)-1640 culture medium (#ZQ-230) was from Zhong Qiao Xin Zhou Biotechnology (Shanghai, China). Rabbit anti-human phospho-AMPK (#5256) antibody was purchased from Cell Signaling Technology (Danvers, USA), while rabbit anti-mouse polyclonal to AMPK antibody (#ab194920) was purchased from Abcam (Cambridge, USA). Radio Immunoprecipitation Assay Lysis Buffer and Tris buffered saline-Tween were from SAINT-BIO (T10272, Shanghai, China). The anti-mouse Scavenger receptor-A (SR-A, #ab314227) antibody was from Abcam (Cambridge, USA), while the anti-SR-A human antibody (#WH0004481M1) was from Sigma-Aldrich (Saint Louis, USA). β-actin monoclonal antibody (#PMC201) and horseradish peroxidase (HRP) anti-rabbit immunoglobulin G (IgG) antibody (#PMS302) were purchased from Proteinbio Biotechnology (Nanjing, China). Capture anti-myeloperoxidase (MPO) (#AF3667, R&D Systems, Minneapolis, MN, USA), the secondary mouse HRP-conjugated anti-DNA antibody (#1154467501, Roche, Indianapolis, IN, USA) and the 3,3′,5,5’-Tetramethylbenzidine substrate (#34021, Thermo Scientific, Hudson, New Hampshire, USA) were purchased for MPO-DNA complex detection.

### Mice

Four- to six-week-old BALB/c male nude mice (16–18 g) were obtained from Shanghai Bikai Keyi Biotechnology with animal license number SCXK (Shanghai) 2018-0006. Animals were subjected to standard pathogen-free conditions and provided with standard pellet feed and a free water supply. The mice underwent adaptive feeding for 3 days before the experiment. The treatment of the experimental mice meets the requirements of animal ethics and has been approved by Lianyungang Oriental Hospital affiliated to Xuzhou Medical University Institutional Animal Ethics Committee (no. 2023-028-01).

### Tumor-bearing nude mice model construction and treatment

KYSE150 cells, which were in a good growth state and were in a vigorous growth period, were resuspended to form a cell suspension at a concentration of 1 × 10^7^ cells/ml. Forty-four BALB/c nude mice were randomly selected. Under sterile conditions, the left abdominal cavity of nude mice was injected with 0.2 ml of KYSE150 cell suspension. Tumor formation was monitored by bioluminescence. When the tumor grew to around 150 mm^3^, 40 nude mice with similar tumor sizes were selected for further treatment. These mice were randomly divided into four groups: sham, radiation, ozone and radiation+ozone group (*n* = 10 per group).

The Sham group received no treatment. The radiation group received 8 grays (Gy) of radiation at a dose rate of 0.86 Gy/min using an RS 2000 PRO X-ray irradiator (Rad Source Technologies, USA). Only the tumor locations were exposed to radiation, while other parts of the mouse body were shielded with lead plates. The ozone treatment group received only intraperitoneal injections of fresh ozone (30 μg/ml) for treatment, with 0.5 ml per mouse for 13 consecutive days. The combined group (radiation+ozone) received 8 Gy of radiation after intraperitoneal injection of fresh ozone (30 μg/ml) for treatment. Each mouse received 0.5 ml for 13 consecutive days.

The tumor volume, as well as body weight, of each mouse were monitored every other day by caliper measurements. Half of the mice in each group were anesthetized (200 mg/kg ketamine, 10 mg/kg xylazine), blood was withdrawn by cardiac puncture 2 hours after 13 days of treatment and tumor tissues and blood (by either orbital eye bleed or cardiac puncture) were collected. Other mice were fed normally. Tumor suppression rates (TSR), absolute growth delay (AGD), normalized growth delay (NGD) and enhancement factor (EF) were calculated according to previously documented methods [20, 21]. The mice were subjected to euthanasia during the experiment if tumor volume/weight reached >10% of their body weight or when the experiments were completed.

### Peripheral cell-free DNA (cf-DNA) detection

Cf-DNA detection was introduced according to the Quant-iT™ PicoGreen® dsDNA Assay Kit (Thermo Fisher Scientific, Carlsbad, CA, USA) instructions. First, Quant-iT™ PicoGreen® reagent was diluted with 1 × Tris-ethylenediaminetetraacetic acid buffer 200 times to prepare a working solution. One milliliter of peripheral blood was collected from mice and centrifuged at 1400 g (4°C, 15 minutes) in order to collect serum. Each well of the enzyme-linked plate was filled with 50 μl of serum and 50 μl of working solution. The mixture on the plate was carefully shaken and mixed well before a 5-minute reaction in the dark. The fluorescence intensity was detected using a MD/SpectraMax M2e plate reader (Molecular Devices, Sunnyvale, CA, USA) at 480/520 nm, which is proportional to the cf-DNA content.

### Serum cytokine analysis

According to the respective manufacturer’s instructions, measurement of cytokine levels, including interleukin-6 (IL-6), interferon-γ (IFN-γ), tumor necrosis factor-α (TNF-α) and matrix metalloproteinase-9 (MMP-9), was completed using commercial ELISA kits. MPO-DNA complexes were detected in the mouse serum using MPO (1:200), the secondary mouse anti-DNA-POD (1:500) and TMB substrate as previously described [22].

### Immunohistochemistry

Mouse tumor tissue was collected, fixed and then embedded in paraffin. The paraffined tumor was subjected to cutting into 5-μm-thick sections (HistoCore multicut, Leica Biosystems, Wetzlar, Germany). After conventional dewaxing, the slices were subjected to antigen extraction in 10 mM citrate buffer (pH 6.0, 90°C). At 4°C, the slices were diluted with anti-P-AMPK or anti-SR-A polyclonal antibodies at 1:100 and incubated for 1 hour with HRP anti-rabbit IgG antibodies (room temperature). Then, the slides were washed, followed by two-step staining with diaminobenzidine and hematoxylin. The test was under a microscope (Olympus BX-41; Olympus, Tokyo, Japan).

### Western blotting

Peripheral serum (50 μl) was diluted with 50 μl of radioimmunoprecipitation assay buffer and placed on ice to stand for 15 minutes. Then, the mixture was mixed evenly and centrifuged at 3000 rpm for 15 minutes. The obtained supernatant was transferred to another clean Eppendorf tube. Five microliter supernatant solution was used for protein quantification using the BCA method. The remaining protein solutions were added to the loading buffer and boiled in water for 15 minutes. The samples (20 μl) were added to each lane for electrophoresis. The obtained protein was then transferred to the polyvinylidene fluoride membrane, sealed with blocking solution for 1 hour and washed three times with Tris-buffered saline-Tween buffer (10–15 minutes each time). Subsequently, an overnight incubation was conducted for the PVDF membrane, along with primary antibodies (p-AMPK antibody and SR-A antibody, both 1:1000) at 4°C. Then, a 3-hour incubation with HRP anti-rabbit IgG antibiotics (1:2000) was conducted for the PVDF (room temperature). Finally, PVDF membrane was stained and placed in the iBright CL750 Imaging System (Invitrogen, Carlsbad, CA, USA) to detect the indicated proteins.

### Statistical analysis

We used Graphpad Prim9 statistical software for data analysis and graphic creation. The comparisons used a *t*-test, or one-way or two-way analysis of variance (Tukey’s multiple comparisons test). *P* < 0.05 was defined as statistical significance.

## RESULTS

### Ozone enhanced sensitivity of the KYSE150-transplanted nude mouse model for esophageal cancer to radiotherapy

**Fig. 1:**
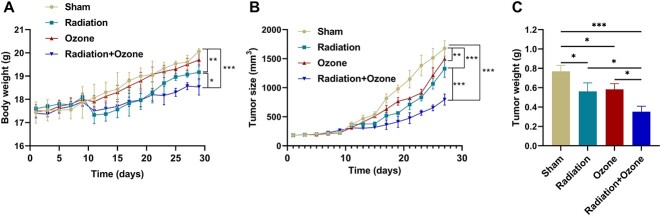
Radiosensitivity effect of ozone therapy on the KYSE150-transplanted nude mouse tumor model of esophageal cancer. (A) The average body weight of mice in each group. (B) The tumor volume size of mice in each group. (C) The tumor weight of each group. ^*^P < 0.05, ^**^P < 0.01, ^***^P < 0.001.

The radiation group and radiation+ozone group showed inhibitory effects on mouse body weight (Fig. 1A). The radiation group, ozone group and radiation+ozone group showed significant inhibitory effects on mouse tumor growth versus the sham group (Fig. 1B). By weighing the tumor, the weight of the tumor in the radiotherapy group, ozone group and radiation combined ozone group was significantly lower than that in the sham group. Moreover, the radiation combined with ozone group mice showed lower tumor weight than the radiation and ozone group mice (Fig. 1C). Based on the calculation of TSR for the sham group, it was found that the TSRs for the radiation group, ozone treatment group and radiation combined ozone group were 56.2%, 64.1% and 76.7%, respectively (Table 1). Compared with the doubling time of the sham group (11.4 ± 1.4 days), those of the radiotherapy group (15.2 ± 1.8) and the ozone treatment group (13.8 ± 1.6 days) were significantly prolonged, as well as the mice with the radiotherapy combined with the ozone treatment group (19.6 ± 1.8 days) (Table 1). Moreover, ozone enhanced the sensitivity of KYSE150-transplanted tumors to radiation therapy, with an EF of 1.53 (Table 1).

**Table 1:** Sensitization effect of ozone on radiation therapy in esophageal cancer transplanted tumor model

Groups	TSR (%)	Doubling time (days)	AGD (d)	NGD (d)	EF
Sham		11.4 ± 1.4			
Radiation	56.2	15.2 ± 1.8	3.8		
Ozone	64.1	13.8 ± 1.6	2.4		
Radiation+Ozone	76.7	19.6 ± 1.8	8.2	5.8	1.53

Notes: Doubling time = d × lg2/lg(Vd/V0)

(d represented the length of time (days) between two measurements, Vd was the tumor volume at the time after treatment, and V0 was the tumor volume at the start of treatment).

TSR, tumor suppression rate (%) = (average tumor weight of control group − average tumor weight of drug administration group)/average tumor weight of control group×100%.

AGD, absolute growth delay = the doubling time of the treatment group − the doubling time of the control group.

NGD, nominal growth delay = the time for the tumor in the Radiation+Ozone treatment groups to grow to 40 times its initial volume − the time for the tumor in the radiation-only treatment groups to grow to 40 times its initial volume.

EF, enhancement factor = the NGD of Radiation+Ozone treatment group/the AGD of the radiation group.

### The effect of ozone on the levels of NETs and cytokines in peripheral blood of tumor model mice

**Fig. 2:**
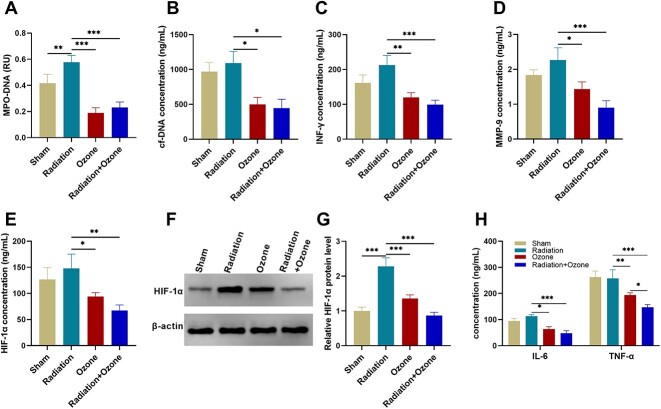
The effect of ozone on the circulating levels of NETs and cytokines in esophageal cancer-modeling mice. (A) The level of MPO-DNA complexes. (B) The level of cf-DNA. (C) The level of IFN-γ. (D) The level of MMP-9. (E–G) The level of HIF-1α. (H) The level of TNF-α/IL-6. ^*^P < 0.05, ^**^P < 0.01, ^***^P < 0.001.

MPO-DNA complex, an indicative factor for NETs, was quantified. The results showed an increase in MPO-DNA complex after radiation but decreased if ozone was used to intervene (*P* < 0.01, Fig. 2A). The levels of cf-DNA (*P* < 0.05, Fig. 2B) were also decreased if ozone was used to intervene. NETs can cause severe inflammatory reactions in esophageal cancer [23]. We used the ELISA detection method or western blot assays to detect the levels of cf-DNA, IFN-γ, TNF-α, IL-6, HIF-1α and MMP-9 in the peripheral blood of mice. Compared with the radiation group mice, the ozone treatment group and the radiation combined ozone treatment group mice showed lower levels of IFN-γ (*P* < 0.01, Fig. 2C) and MMP-9 (*P* < 0.05, Fig. 2D). Serum HIF-1α level (*P* < 0.05, Fig. 2E) and tumor HIF-1α protein level (*P* < 0.001, Fig. 2F and G) were reduced after ozone treatment or the radiation+ozone treatment compared to those after radiation. Serum TNF-α/IL-6 levels were also decreased after ozone treatment or radiation+ozone treatment compared to those after radiation (*P* < 0.05, Fig. 2H).

### The protein levels of p-AMPK and SR-A in tumor tissue of mice

**Fig. 3:**
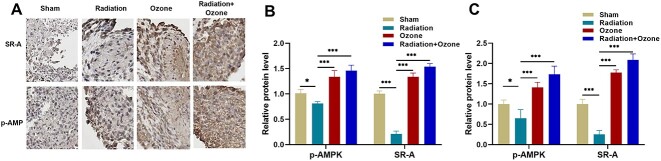
The effects of ozone intervention on the protein levels of p-AMPK and SR-A. (A) Representative immunocytochemical images of p-AMPK and SR-A in mouse tumor samples. Scale bar  =  20 μm. (B) Statistical histograms of the Western blot assay for the protein levels of p-AMPK and SR-A in mouse serum. (C) Statistical histograms of the Western blot assay for the protein levels of p-AMPK and SR-A in mouse tumor tissues. ^*^P < 0.05, ^**^P < 0.01, ^***^P < 0.001.

For exploration of the potential mechanism in ozone-inhibiting NET generation, we measured the protein levels of p-AMPK and SR-A in mouse tumor tissues using immunohistochemistry and Western blot analyses. The immunohistochemical results revealed a decrease in the expression of p-AMPK and SR-A proteins in the radiation group, compared with the sham group, but increases in the ozone treatment group, especially in the ozone+radiation group (Fig. 3A). Western blotting analysis showed that ozone upregulated the AMPK-SR-A pathway proteins in mouse serum, including p-AMPK and SR-A proteins (*P* < 0.05, Fig. 3B and Supplementary Fig. 1A). In tumor tissue, p-AMPK and SR-A proteins were increased after ozone treatment or the radiation+ozone treatment compared to those after radiation (*P* < 0.05, Fig. 3C and Supplementary Fig. 1B). These results allowed us to speculate that ozone can promote AMPK phosphorylation, thereby upregulating SR-A to phagocytose NETs, ultimately reducing the generation of radiation resistance.

## DISCUSSION

Radiation therapy is one of the mainstream traditional treatments for cancer today. When combined with other treatments, it is particularly effective in controlling certain tumors [24]. In theoretical research, radiation therapy has an inhibitory effect on almost all tumor cells. However, in practical applications, different sensitivity of cells to radiation can lead to different therapeutic effects [25]. Despite the development of new radiation therapy technologies, radiation resistance still leads to radiotherapy failure, cancer metastasis, cancer recurrence and poor prognosis. Radiation resistance remains the main obstacle to improving treatment outcomes [26]. Radiotherapy resistance may occur in multiple ways, involving complex mechanisms due to its heterogeneity with tumors and the surrounding microenvironment, as well as many interactions with genetic changes [26]. Therefore, it is necessary to develop new sensitization methods to achieve better cancer suppression effects with radiation therapy. Biomolecule ozone has been found to inhibit the growth of various carcinoma cells and completely retard rabbit squamous cell carcinomas [14, 27]. In this study, apart from the anticancer effect of irradiation, we observed that ozone had an *in vivo* inhibitory effect on esophageal cancer. Notably, we identified ozone as a sensitization factor for esophageal cancer radiosensitivity. We found that ozone can alter NETs, thereby enhancing the radiation therapy effect of esophageal cancer xenografts.

NETs are a network structure composed of DNA protein complexes secreted by activated N2-type tumor-associated proteins, embedded with various active proteins, including MMP9 [7]. In terms of tumor response to radiation therapy, the NET-dependent mechanism of cancer pathology has begun to receive attention. Radiation exposure can cause neutrophil death, leading to the production of NETs [28]. Increasing studies have confirmed the role of NET in cancer resistance [29]. In this study, we first confirmed that ozone therapy interventions can make the tumor body formed by esophageal cancer cells more sensitive to radiation. Then, we measured the serum cf-DNA levels of mice, which can reflect the effect of ozone on the expression of NETs. Compared with mice that only received radiation therapy, mice that received ozone therapy alone or in combination significantly reduced their serum cf-DNA levels. Radiation therapy can trigger the release of pro-inflammatory mediators and increase tumor-infiltrating immunostimulatory cells [30]. Here, we found that ozone therapy alone or in combination with radiation therapy significantly reduced the levels of INF-γ, MMP-9, IL-6, TNF-α and HIF-1α. This indicates that ozone has a relieving effect on the NETs formed by radiation therapy.

Circulating NET levels have been identified to be elevated in advanced esophageal cancer patients [23]. AMPK, a serine threonine protein kinase, has demonstrated a positive interconnectedness with NETs [31]. Its activation can promote macrophage phagocytosis of HMGB1 and increase the clearance rate of NETs [19]. SR-A is important for canonical NET formation via selective ERK phosphorylation. It has been reported that ozone reduces peritoneal fluid production in H22 tumor-bearing mice by promoting phagocytic damage-related molecular patterns and reducing the production of NETs by activating AMPK and upregulating SR-A [32]. Ozone therapy can activate AMPK/SOCS3, relieving chemotherapeutic enteritis [19]. Regarding the radio-sensitization mechanism of ozone in esophageal cancer, we found that ozone activates AMPK and increases SR-A protein levels. These indicate that ozone induces SR-A production by activating AMPK phosphorylation, thereby reducing the formation of NETs. This indicates that ozone can serve as a new method of treating esophageal cancer in combination with radiation therapy.

In summary, our study showed that ozone exhibited a significant sensitization effect in radiation therapy in the KYSE150-bearing mouse model of esophageal cancer. Ozone may reduce the formation of NETs through the AMPK/SR-A pathway, thereby reducing tumor growth. This study provides insights into ozone therapy for radiosensitization of esophageal cancer. Further research is needed to fully elucidate the potential molecular mechanism of ozone’s role in the radiosensitization of human esophageal cancer.

## AUTHOR CONTRIBUTIONS

Conceptualization: Jiayou Guo, Jiayi Guo, Jianxin Ma; Methodology: Jiayou Guo, Jiayi Guo, Beibei Cheng; Formal analysis and investigation: Mengxiao Gong, Xingbang Sun, Hongwei Zhang; Writing—original draft preparation: Jiayou Guo, Jiayi Guo; Writing—review & editing: Jianxin Ma; Resources: Beibei Cheng, Mengxiao Gong; Supervision: Jianxin Ma. All authors reviewed the results and approved the final version of the manuscript.

## CONFLICT OF INTEREST

The authors declare that they have no conflicts of interest to report regarding the present study.

## FUNDING

The authors received no specific funding for this study.

## ETHICS APPROVAL

This experiment was conducted with the approval of the Animal Ethics Committee of Lianyungang Oriental Hospital affiliated to Xuzhou Medical University (no. 2023-028-01). All animal experiments in this study were in strict accordance with the protocols stated in the Guide for the Care and Use of Laboratory Animals published by the US National Institutes of Health. Appropriate measures were taken to minimize the number and suffering of animals.

## DATA AVAILABILITY

All data generated or analyzed during this study are included in this article. Further inquiries can be directed to the corresponding author.

## Supplementary Material

Supplementary_Figure_1_rrae041
