# Supplementary figures and images for: Ozone enhances the efficacy of radiation therapy in esophageal cancer
Source: J Radiat Res. 2024 Jun 6;65(4):467–73. doi: 10.1093/jrr/rrae041 (PMC11262864; doi:10.1093/jrr/rrae041)

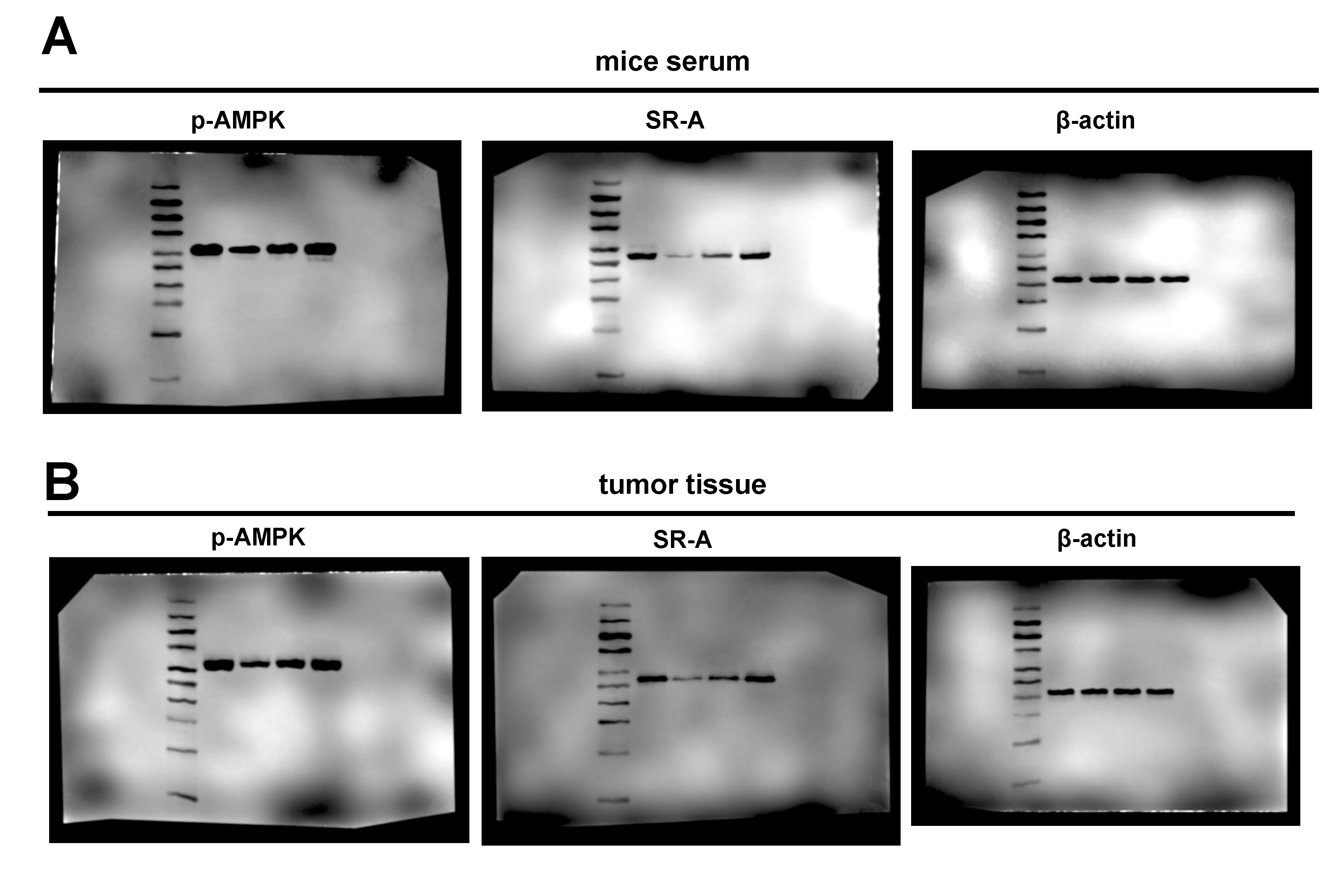

Supplement: Supplementary_Figure_1_rrae041 [file supplementary_figure_1_rrae041.jpeg]
